# Efficacy of Duloxetine in Patients with Chronic Pain Conditions

**DOI:** 10.2174/157488511798109592

**Published:** 2011-11

**Authors:** Vladimir Skljarevski, Shuyu Zhang, Smriti Iyengar, Deborah D’Souza, Karla Alaka, Amy Chappell, Joachim Wernicke

**Affiliations:** Lilly Research Laboratories, Indianapolis, IN 46285, USA

**Keywords:** Chronic low back pain, chronic pain, diabetic peripheral neuropathic pain, duloxetine, efficacy, fibromyalgia, osteoarthritis.

## Abstract

The primary objective of this study is to review the efficacy of duloxetine in treating chronic pain using the Initiative on Methods, Measurement, and Pain Assessment in Clinical Trials (IMMPACT) recommendations for clinical significance across chronic pain states. These include pain intensity, patient ratings of overall improvement, physical functioning, and mental functioning. This review comprised the side-by-side analyses of 12 double-blind, placebo-controlled trials of duloxetine in patients with chronic pain (diabetic peripheral neuropathic pain, fibromyalgia, chronic pain due to osteoarthritis, and chronic low back pain). Patients received duloxetine (60 to 120 mg/day) or placebo. Average pain reduction was assessed over 3 months as the primary efficacy outcome. Other measures used were physical function and Patient Global Impression of Improvement. In 10 of the 12 studies, statistically significant greater pain reduction was observed for duloxetine- compared with placebo-treated patients. The response rates based on average pain reduction, improvement of physical function, and global impression were comparable across all 4 chronic pain states. Compared with patients on placebo, significantly more patients treated with duloxetine reported a moderately important pain reduction (≥30% reduction) in 9 of the 12 studies, a minimally important improvement in physical function in 8 of the 12 studies, and a moderately important to substantial improvement in Patient Global Impression of Improvement rating in 11 of the 12 studies. The analyses reported here show that duloxetine is efficacious in treating chronic pain as demonstrated by significant improvement in pain intensity, physical functioning, and patient ratings of overall improvement.

## INTRODUCTION

The prevalence of chronic pain in the United States and Europe has been estimated at 35.5% [1] and 19% (of adults with moderate to severe intensity) [2], respectively. Depending on how it is defined, chronic pain is estimated to have a worldwide prevalence of 11% to 55% [3]. Pain represents the most common reason for patients to seek medical counsel [4]. Many types of pain, including chronic pain, affecting different patient populations (elderly, children, minorities, substance abusers) remain undertreated [5,6], and a complete resolution of pain is rarely achieved [7]. Untreated or undertreated pain has significant physical, psychological, social, and financial consequences [8].

Unlike acute (nociceptive) pain, chronic pain is a pathological state associated with functional and structural changes within the peripheral and central nervous systems. Among these changes, central sensitization and impairment of associated pain inhibitory circuits have been extensively researched [9-12]. These pathophysiological mechanisms are involved in and at least partially responsible for the development and maintenance of chronic pain states, regardless of their respective underlying etiologies (e.g., neuropathy, inflammation, or tissue damage).

Three main categories of chronic pain are typically recognized [13]. These include neuropathic pain (resulting from nerve damage or dysfunction either in the peripheral or in the central nervous system, e.g., diabetic peripheral neuropathic pain [DPNP]), inflammatory/joint-related pain (resulting from peripheral inflammation or peripheral tissue/joint damage, e.g., early-stage osteoarthritis), and noninflammatory/non-neuropathic pain (also called functional pain by some pain researchers) which results from centrally impaired pain processing like in fibromyalgia (FM). Chronic pain conditions such as chronic low back pain (CLBP) may result from a number of causes, which can technically fall into any of these 3 chronic pain categories just discussed.

Serotonin (5-HT) and norepinephrine (NE) have been implicated in the mediation of endogenous pain inhibitory mechanisms *via *the descending pain inhibitory pathways in the brain and spinal cord [12, 14, 15]. In chronic pain states, the net inhibitory effect of these monoamines is postulated to be reduced or lost; consequently, shifting the descending pain modulatory system from a state of inhibition towards a state of pain facilitation [16, 17]. Duloxetine is a potent and selective inhibitor of 5-HT and NE reuptake *in vitro* and *in vivo* in the central nervous system (CNS) [18]. Preclinical studies have shown that duloxetine effectively reduces pain behavior across a range of persistent, neuropathic, and inflammatory pain models [19-21], in a dose range that is consistent with inhibition of 5-HT and NE reuptake. Thus, the analgesic effect of duloxetine is believed to result from increased activity of 5-HT and NE within the CNS [18, 20, 21], presumably either by enhancing descending pain inhibitory pathways in the brain and spinal cord or other unknown CNS actions. 

Irrespective of the underlying pathology or its absence, pain sensation becomes maladaptive, pathological, and enhanced, at least partly, due to the imbalance between excitatory and inhibitory pathways within the CNS. This supports the hypothesis that a centrally active agent impacting the pain processing/sensing pathways may have an analgesic effect across chronic pain conditions. In view of this, duloxetine has been evaluated in DPNP, FM, chronic pain due to osteoarthritis (OA), and CLBP. 

In previously published clinical trials, duloxetine has been shown to be effective in treating patients with DPNP [22-24] and FM [25-28]. Data from placebo-controlled trials have also provided evidence of duloxetine’s efficacy in OA pain [29, 30] as well as CLBP [31, 32].

While prior studies established the superiority of duloxetine relative to placebo in DPNP, FM, OA, and CLBP, the analyses presented here address the clinical significance of those findings. In order to accomplish that, we used the criteria of clinical significance in chronic pain trials as proposed by the Initiative on Methods, Measurement, and Pain Assessment in Clinical Trials (IMMPACT) [33]. These include pain intensity, physical functioning, and patients’ ratings of overall improvement.

Safety and tolerability of duloxetine has been described previously (and will not be addressed here) in the individual publications cited above, and articles with a focus on safety [34-42].

## METHODS

This report is based on the side-by-side analyses of 12 double-blind, placebo-controlled, randomized, multicenter clinical trials of duloxetine in patients with chronic pain (OA: study HMFG [29], study HMEP [30]; CLBP: study HMEN [43], study HMEO [31], study HMGC [32]; DPNP: study HMAW [22], study HMAVb [23], study HMAVa [24]; FM: study HMBO [25], study HMCA [26], study HMCJ [27], study HMEF [28]). The trials were of at least 12-week duration and evaluated duloxetine doses of 60-mg to 120-mg daily. Several of the studies in FM were of 6-month duration, but for comparison purposes only 3-month data are presented here.

Details about the inclusion and exclusion criteria for each of the chronic pain states are provided in the published manuscripts for the individual studies [22-28, 30, 32, 43]. All of the studies required that patients have a 24-hour average pain rating of 4 or greater based on an 11-point numerical rating scale at study entry and have chronic pain for at least 3 to 6 months prior to study entry. In the OA and FM studies, patients had to meet the criteria for OA of the knee or FM as defined by the American College of Rheumatology. In the CLBP studies, patients included were those with non-neuropathic pain (Class 1 and 2 per the Quebec Classification of Spinal Disorders).

Patients were excluded if they had any serious medical or psychiatric condition that could compromise their participation in the study. All protocols excluded patients with clinically significant impairment in mental function. Patients with major depressive disorder were excluded from all studies except those in FM.

The primary efficacy measure in all studies was the 24-hour average pain rating (rating scale ranging from 0 [no pain] to 10 [worst possible pain]). Outcome measures used to assess physical functioning included the Brief Pain Inventory (BPI) average physical interference (used in the DPNP and FM studies, the average of 3 physical interference items, namely: general activity, walking ability, and normal work; physical function response was defined as 1 point decrease on the average of the 3 physical interference items [general activity, walking ability, and normal work]), the Western Ontario and McMaster Universities (WOMAC) [44] physical function subscale (in the OA studies), and the Roland Morris Disability Questionnaire (RMDQ-24) [45] (in the CLBP studies). The Patient Global Impression of Improvement (PGI-I) scale measured patients’ ratings of overall improvement compared to study baseline (range from 0 [very much better] to 7 [very much worse]).

The benchmarks used to assess the clinical importance of changes in some of the above measures were based on the recommendations of the IMMPACT consensus on the clinical significance of change across pain states [33]. For measuring pain intensity, a decrease of ≥30% and ≥50% are considered ‘moderately’ and ‘substantially’ important, respectively [33]. One point decrease in BPI physical interference is considered clinically important. A change of ‘much improved’ (PGI-I endpoint = 2) to ‘very much improved’ (PGI-I endpoint = 1) are considered ‘moderately’ to ‘substantially’ important, respectively, on the Patient Global Impression of Change [33]. For the WOMAC scale, a score of ≤31 is considered to be an acceptable physical symptom state [46], and for the RMDQ [47-49] a change of ≤-3.5 is considered clinically meaningful.

### Statistical Methods

All analyses were conducted on an intent-to-treat (ITT) basis. Treatment effects were evaluated based on 2-sided tests with a significance level of 0.05. No adjustments were made for multiple comparisons. When an average score was computed from individual items, it was calculated from nonmissing values.

For all analyses, baseline refers to the last nonmissing observation at or before the random assignment visit and endpoint refers to the last nonmissing observation in the 3-month treatment phase [last observation carried forward (LOCF)].

For continuous variables, an analysis of covariance (ANCOVA) model was used including baseline value, treatment, and investigator. Type III sum-of-squares for the least-squares mean (LS Mean) was used to assess treatment difference. For the categorical variable, Fisher’s exact test was used to assess the treatment difference. For time-to-first-response analysis, the Kaplan-Meier survival estimate was calculated by treatment group at each time point (Week 1 to Week 13). Patients who did not meet response criteria were considered as right-censored observation (a data point is above a certain value but it is unknown by how much). Treatment difference was assessed through log-rank test.

The term ‘significant’ indicates statistical significance throughout the manuscript. SAS version 9 was used to perform all statistical analyses.

## RESULTS

Among OA and CLBP patients, the majority were Caucasian (86.6%), female (63.4%), had a mean age of 56 years, and a mean duration of study drug exposure of 77.7 days. For DPNP patients, the majority were Caucasian (84.4%), male (56.8%), had a mean age of 60 years, and mean duration of study drug exposure of 77.6 days; whereas, in FM patients, the majority were Caucasian (87.5%), female (94.8%), had a mean age of 50 years, and a mean duration of study drug exposure of 110.2 days. In duloxetine-treated OA and CLBP patients, the most common (≥5%) adverse events were nausea (13.9%), dry mouth (7.0%), constipation (6.9%), insomnia (6.6%), diarrhea (5.7%), dizziness (5.7%), somnolence (5.6%), and fatigue (5.0%). The most common (≥5%) adverse events among duloxetine-treated DPNP patients, were nausea (23.9%), somnolence (15.9%), dizziness (11.0%), diarrhea (9.6%), insomnia (8.9%), fatigue (8.6%), constipation (9.4%), hyperhidrosis (8.5%), dry mouth (7.5%), and decreased appetite (5.3%). Among duloxetine-treated FM patients, the most common (≥5%) adverse events were nausea (29.3%), headache (20.0%), dry mouth (18.2%), insomnia (14.5%), fatigue (13.5%), constipation (14.5%), diarrhea (11.6%), dizziness (11.0%), somnolence (9.6%), hyperhidrosis (6.8%), and decreased appetite (6.5%).

Patients taking duloxetine 60 mg to 120 mg daily, on a group mean level, demonstrated significantly greater reduction in 24-hour average pain compared with placebo in 10 of the 12 chronic pain studies (Table **1**). In the 2 remaining studies, duloxetine was numerically better than placebo. The observed baseline pain severity across the studies was approximately 6. On average, duloxetine-treated patients reported pain reduction of -1.43 to -3.14, while the range for placebo-treated patients was -0.67 to -1.93.

On the level of individual patient response, duloxetine-treated patients reported a significantly higher response rate in 8 of the 12 chronic pain studies based on 30% response criteria and 7 of the 12 studies based on 50% response criteria (Table **2**). On average, the 30% and 50% response rate for duloxetine-treated patients ranged from 37% to 69% and 26% to 53%, respectively, while the range for placebo-treated patients was 27% to 49% and 15% to 35%, respectively.

Compared with patients on placebo, patients treated with duloxetine had a significantly greater response rate in physical function improvement in 8 of the 12 studies (Table **3**).

Compared with patients on placebo, patients treated with duloxetine had a significantly greater PGI-improvement response rate in 11 of the 12 studies (Table **4**).

The survival analysis of the time to first 30% average pain reduction across the 12 CP studies showed a significant separation as early as 1 to 2 weeks, except for 3 studies where significant separation was not achieved until Week 3. Furthermore, the distribution curves of time to response for all 12 studies showed significant separation between duloxetine and placebo during the 3-month treatment phase (P<0.01).

## DISCUSSION

Duloxetine hydrochloride is a selective dual 5-HT and NE reuptake inhibitor with central analgesic properties. Preclinically, duloxetine is efficacious in models of persistent, inflammatory, and neuropathic pain [19-21], suggesting that it may be efficacious in the treatment of chronic pain conditions in which central sensitization is believed to be one of the underlying pathophysiological mechanisms [50, 51]. Central sensitization is dependent upon the activation of a descending pain facilitatory pathway originating in the brainstem [52]. Duloxetine, by enhancing monoaminergic tone, may potentially reduce the consequences of central sensitization by shifting the descending pain modulatory pathway from a state of facilitation to a state of inhibition [16, 17]. Consistent with the preclinical data, duloxetine, has demonstrated remarkable consistency of analgesic effect across all 3 main categories of chronic pain.

Duloxetine-treated patients demonstrated a significantly greater pain reduction compared with placebo-treated patients in 10 of the 12 studies and a numerically greater reduction in the remaining 2 studies.

Patients receiving duloxetine for the treatment of chronic pain states had significantly higher response rates corresponding to clinically moderate and substantially important improvement compared with patients receiving placebo.

A 30% reduction from baseline to endpoint was used as one measure of response. A previous report by Farrar *et al*. [53] estimated that a decrease in pain intensity of 30% was associated with the patient rating of ‘much improved’ and decreases of ≥50% are associated with the patient rating of ‘very much improved’ [54]. A subsequent study [55] has also demonstrated that on a 0 to 10 numeric rating scale of pain intensity for patient-reported ‘average’ and ‘worst’ pain, a percentage change of 34% was best associated with a clinically important difference, namely the PGI-I category of ‘much better’ or higher, and a 51% reduction in pain from baseline was associated with ‘very much better’.

Duloxetine-treated patients demonstrated significant improvement in physical functioning in 8 of the 12 studies. Patients receiving duloxetine showed significant physical-function improvement as assessed by the BPI-Interference, as well as disease-specific measures like WOMAC physical function subscale (OA), and RMDQ-24 (CLBP). Regardless of the measures used in these studies, patients with chronic pain had a significant improvement in overall physical functioning.

There was also a significant overall improvement in duloxetine-treated patients as demonstrated by the PGI-improvement rating (a measure of the degree of change at the time of assessment).

The time-to-response data demonstrated that there was a significant separation between duloxetine and placebo at 1 to 2 weeks for 9 of the 12 studies and at Day 21 for the remaining 3 studies.

The limitations to this work should be noted. The results are based on 3-month long trials and the results may not be extrapolated to longer treatment durations. The results of these studies also may not generalize to all individuals with chronic pain conditions, since patients with certain comorbidities were excluded from the studies.

Based on the study design for each of the individual studies, the time-to-onset of significant pain relief may not be comparable. However, in a majority of the studies, the time-to-onset of response is 1 to 2 weeks.

Even though IMMPACT recommends assessing mental functioning as part of the clinical response using Beck Depression Inventory (BDI) in clinical trials, this paper did not include such analyses because BDI was only collected in 8 of the 12 studies. These studies excluded patients with clinically significant impairment in mental function, and, in addition, patients with major depressive disorder (MDD) were excluded from all studies except those in FM. As a result, the BDI total scores at baseline were very low (ranges from 4.29 to 7.33 for studies that excluded MDD) at the outset and consequently there was not much room for improvement.

In summary, the analyses reported here show that duloxetine is efficacious in treating four distinctively different chronic pain conditions, as demonstrated by clinically significant improvement in pain severity, physical functioning, and patients’ ratings of overall improvement. The overall pattern and magnitude of response were comparable across the 4 chronic pain conditions, suggesting that duloxetine is an effective centrally-acting general analgesic.

## Figures and Tables

**Table 1.** Summary of 24-Hour Average Pain Rating (3-Month Results) Across All Chronic Pain Studies of Duloxetine

Indication	Study	Treatment	Mean Change (SE)

OA	HMFG	Placebo	-1.72 (0.18)
		Duloxetine 60 – 120 mg	-2.51 (0.20)***

	HMEP	Placebo	-1.93 (0.18)
		Duloxetine 60 – 120 mg	-2.64 (0.19)**

CLBP	HMEN	Placebo	-1.45 (0.21)
		Duloxetine 60 – 120 mg	-2.09 (0.21)*

	HMEO	Placebo	-1.82 (0.20)
		Duloxetine 60 mg	-2.27 (0.20)
		Duloxetine 120 mg	-2.21 (0.20)

	HMGC	Placebo	-1.65 (0.15)
		Duloxetine 60 mg	-2.25 (0.15)**

DPNP	HMAW	Placebo	-1.69 (0.24)
		Duloxetine 60 mg	-2.86 (0.24)***
		Duloxetine 120 mg	-3.14 (0.24)***

	HMAVa	Placebo	-1.39 (0.23)
		Duloxetine 60 mg	-2.72 (0.22)***
		Duloxetine 120 mg	-2.84 (0.23)***

	HMAVb	Placebo	-1.60 (0.18)
		Duloxetine 60 mg	-2.50 (0.18)***
		Duloxetine 120 mg	-2.47 (0.18)***

Fibromyalgia	HMBO	Placebo	-0.67 (0.22)
		Duloxetine 120 mg	-1.43 (0.22)*

	HMCA	Placebo	-1.16 (0.21)
		Duloxetine 60 mg	-2.39 (0.22)***
		Duloxetine 120 mg	-2.40 (0.22)***

	HMCJ	Placebo	-1.39 (0.20)
		Duloxetine 60 mg	-1.99 (0.20)*
		Duloxetine 120 mg	-2.31 (0.20)***

	HMEF	Placebo	-1.17 (0.19)
		Duloxetine 60 mg	-1.50 (0.20)

Abbreviations: OA-osteoarthritis, CLBP-chronic low back pain, DPNP-diabetic peripheral neuropathic pain, SE-standard error.

* P<.05 versus placebo,

** P<.01 versus placebo,

*** P<.001 versus placebo.

**Table 2.** Summary of 30% and 50%Average Pain Rating (3-Month Results) Across All Chronic Pain Studies of Duloxetine

Indication	Study	Treatment	30%Response Rate, (%)	50%Response Rate, (%)

OA	HMFG	Placebo	44.1	32.3
		Duloxetine 60 – 120 mg	65.3***	43.8

	HMEP	Placebo	44.5	29.4
		Duloxetine 60 – 120 mg	59.3*	47.2**

CLBP	HMEN	Placebo	40.0	27.0
		Duloxetine 60 – 120 mg	53.2	38.5

	HMEO	Placebo	43.4	29.2
		Duloxetine 60 mg	53.6	34.5
		Duloxetine 120 mg	57.8*	36.7

	HMGC	Placebo	48.7	34.7
		Duloxetine 60 mg	56.9	48.7**

DPNP	HMAW	Placebo	33.0	26.0
		Duloxetine 60 mg	56.0***	49.0***
		Duloxetine 120 mg	56.0***	52.0***

	HMAVa	Placebo	41.5	27.0
		Duloxetine 60 mg	62.7**	43.0*
		Duloxetine 120 mg	69.4***	53.0***

	HMAVb	Placebo	43.4	30.0
		Duloxetine 60 mg	68.1***	50.0
		Duloxetine 120 mg	64.0**	39.0

Fibromyalgia	HMBO	Placebo	26.5	14.7
		Duloxetine 120 mg	38.0	26.0*

	HMCA	Placebo	33.0x	23.0
		Duloxetine 60 mg	55.0***	41.0**
		Duloxetine 120 mg	Duloxetine 120 mg	41.0**

	HMCJ	Placebo	36.0	23.7
		Duloxetine 60 mg	50.7*	34.0
		Duloxetine 120 mg	52.1**	40.1**

	HMEF	Placebo	31.7	25.1
		Duloxetine 60 mg	37.3	29.1

Abbreviations: OA-osteoarthritis, CLBP-chronic low back pain, DPNP-diabetic peripheral neuropathic pain, %-percent.

* P<.05 versus placebo,

** P<.01 versus placebo,

*** P<.001 versus placebo.

**Table 3.** Summary of Physical Function Response Rate Analysis (3-Month Results) Across All Chronic Pain Studies of Duloxetine

Indication	Study	Treatment	%

OA: Response rate based on WOMAC score	HMFG	Placebo	55.9
		Duloxetine 60 – 120 mg	72.4*

	HMEP	Placebo	40.9
		Duloxetine 60 – 120 mg	71.3***

CLBP: Response rate based on RMDQ score (Change ≤-3.5)	HMEN	Placebo	28.6
		Duloxetine 60 – 120 mg	40.4

	HMEO	Placebo	25.0
		Duloxetine 60 mg	38.6
		Duloxetine 120 mg	39.8

	HMGC	Placebo	33.0
		Duloxetine 60 mg	41.6

DPNP: Response rate based on BPI average physical interference (average of physical interference items: general activity, walking ability & normal work)	HMAW	Placebo	59.8
		Duloxetine 60 mg	68.1
		Duloxetine 120 mg	72.5

	HMAVa	Placebo	61.5
		Duloxetine 60 mg	68.5
		Duloxetine 120 mg	78.5**

	HMAVb	Placebo	56.9
		Duloxetine 60 mg	68.5
		Duloxetine 120 mg	76.9**

Fibromyalgia: Response rate based on BPI average physical interference (average of physical interference items: general activity, walking ability & normal work	HMBO	Placebo	38.2
		Duloxetine 120 mg	57.4**

	HMCA	Placebo	51.7
		Duloxetine 60 mg	71.6**
		Duloxetine 120 mg	65.8*

	HMCJ	Placebo	54.7
		Duloxetine 60 mg	66.7
		Duloxetine 120 mg	73.9***

	HMEF	Placebo	46.1
		Duloxetine 60 mg	58.9*

Abbreviations: OA-osteoarthritis, CLBP-chronic low back pain, DPNP-diabetic peripheral neuropathic pain, %-percent, WOMAC- Western Ontario and McMaster Universities, RMDQ- Roland Morris Disability Questionnaire, BPI-Brief Pain Inventory.

* P<.05 versus placebo,

** P<.01 versus placebo,

*** P<.001 versus placebo.

**Table 4.** Summary of PGI-Improvement Response Rate Analysis (3-Month Results) Across All Chronic Pain Studies of Duloxetine

Indication	Study	Treatment	%

OA	HMFG	Placebo	29.1
		Duloxetine 60 – 120 mg	42.3*

	HMEP	Placebo	40.7
		Duloxetine 60 – 120 mg	61.3**

CLBP	HMEN	Placebo	25.0
		Duloxetine 60 – 120 mg	45.9***

	HMEO	Placebo	38.6
		Duloxetine 60 mg	55.6*
		Duloxetine 120 mg	52.3*

	HMGC	Placebo	32.7
		Duloxetine 60 mg	46.4**

DPNP	HMAW	Placebo	31.5
		Duloxetine 60 mg	57.7***
		Duloxetine 120 mg	58.7***

	HMAVa	Placebo	32.4
		Duloxetine 60 mg	58.0***
		Duloxetine 120 mg	63.6***

	HMAVb	Placebo	29.5
		Duloxetine 60 mg	52.3***
		Duloxetine 120 mg	48.6**

Fibromyalgia	HMBO	Placebo	25.3
		Duloxetine 120 mg	36.8

	HMCA	Placebo	21.6
		Duloxetine 60 mg	43.0***
		Duloxetine 120 mg	45.9***

	HMCJ	Placebo	23.7
		Duloxetine 60 mg	35.7*
		Duloxetine 120 mg	43.7***

	HMEF	Placebo	18.0
		Duloxetine 60 mg	28.3*

Abbreviations: OA-osteoarthritis, CLBP-chronic low back pain, DPNP-diabetic peripheral neuropathic pain, %-percent.

* P<.05 versus placebo,

** P<.01 versus placebo,

*** P<.001 versus placebo.
